# Fear of movement and competence frustration mediate the relationship between pain catastrophising and physical function in people living with axSpA: an online cross-sectional survey

**DOI:** 10.1007/s00296-024-05557-w

**Published:** 2024-03-20

**Authors:** Peter C. Rouse, Thomas Ingram, Martyn Standage, Raj Sengupta

**Affiliations:** 1 Centre for Motivation and Health Behaviour Change, Department for Health, University of Bath, Bath, BA2 7AY UK; 2 Royal National Hospital for Rheumatic Diseases, Royal United Hospitals NHS Foundation Trust, Combe Park, Bath, Avon BA1 3NG UK; 3 Department of Pharmacy and Pharmacology, University of Bath, Bath, BA2 7AY UK

**Keywords:** Surveys and questionnaires, Axial Spondyloarthritis, Physical function, Fear-avoidance, Pain, Competence

## Abstract

The objective of this study is to examine the contribution of pain catastrophising to Axial Spondyloarthritis (axSpA) patient’s physical function and to test the mediating role of fear of movement, and uniquely, the contribution of competence frustration to the fear-avoidance model. Participants (*N* = 98, 70% female, M age = 45.62, SD 12.16) completed an online survey (December 2020–May 2021) distributed in the United Kingdom via the National Axial Spondyloarthritis Society (*n* ≈ 3500; NASS, 2019). The PROCESS SPSS macro was used to test three mediation models using percentile bootstrap 95% confidence intervals (PBCI). A significant indirect effect on the relationship between pain and physical function via fear of movement (*β* = 0.10, 95% PBCI = 0.030–0.183) was observed (Model 1). Model 2 showed the relationship between pain catastrophising and physical function to be significantly mediated by fear of movement (*β* = 0.16, 95% PBCI = 0.005–0.322). Finally, Model 3 showed a significant indirect effect on the relationship between pain catastrophising and physical function via competence frustration (*β* = 0.15, 95% PBCI = 0.014–0.309) but not through fear of movement (*β* = 0.062, 95% PBCI = − 0.134 to 0.248). To our knowledge, this is the first study to examine and demonstrate the unique contribution of competence need frustration to the Fear-avoidance model in people that live with axSpA. Identifying modifiable factors that contribute to disease outcomes such as physical function can improve the care and quality of life for people living with a disease currently without a cure.

**Supplementary Information:**

The online version contains supplementary material available at 10.1007/s00296-024-05557-w.

## Introduction

Axial spondyloarthritis (axSpA) typically affects the axial skeleton and sacroiliac joints causing patients to experience pain, fatigue, reduced movement, and impaired physical function which subsequently compromises their ability to work as well as their health-related quality of life [1, 2]. Treatment options that help axSpA patients to maintain physical function, reduce pain and stay in work are crucial to maintain health-related quality of life and reduce financial burden for people living with axSpA and the National Health Service. [3]. The World Health Organisation (WHO), Assessment of SpondyloArthritis Society (ASAS), International Classification of Functioning (ICF) Core set Recommendations emphasise the important role of personal and environmental factors that underly the axSpA disease process [4].

### Benefits of activity for physical function

Activity and stretching are a cornerstone of treatment for people living with axSpA with evidenced benefits including improved mobility and physical function, reductions in disease activity, and enhanced psychological health [5–8]. However, the majority of people living with axSpA are not sufficiently active to gain these treatment benefits [9, 10]. One factor that has been increasingly acknowledged to contribute to activity intolerance and functioning in the context of chronic pain, and axSpA specifically, is fear avoidance behaviour [11–14]. Despite the prevalence of pain for people living with axSpA, little research has established whether pain-related fear of movement contributes to a reduction in physical function in this population [15].

### Fear-avoidance model

**Fig. 1:**
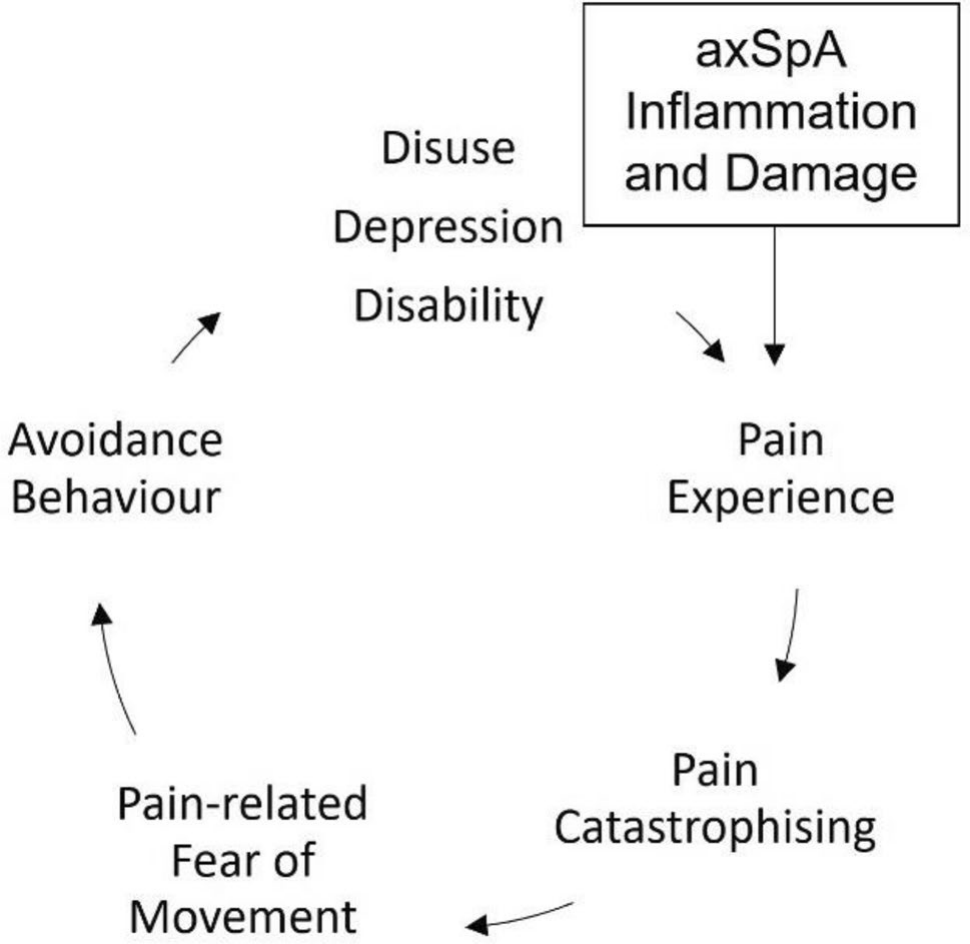
An adapted version of the fear-avoidance model of pain for people living with axSpA [13]

The fear-avoidance model (FAM; Fig. 1) [16] proposes that catastrophising upon pain, that is associated with movement, leads to pain-related fear of movement and subsequently avoidance of activity, leading to disuse and disability. A systematic review of 63 studies in people suffering from chronic musculoskeletal pain, showed fear of movement to be associated with less participation in physical activity and greater levels of pain disability [17].

### Pain-related fear of movement in people living with axSpA

The lack of evidence investigating the fear-avoidance model in people living with axSpA is surprising given that back pain is a leading symptom experienced [18]. Recent research has identified that people living with axSpA do experience pain-related fear of movement but there is mixed evidence regarding the predictive utility of fear of movement for indices of physical function, physical activity, and disease activity [12, 13, 19, 20]. Further, few studies have tested the relationship between fear of movement and indices of physical function in people living with axSpA. One exception is an observational study of 173 Belgian axSpA patients in which the predictive utility of fear of movement was tested, on key outcome measures in axSpA including activity limitation, and disease activity [13]. Results revealed that fear of pain consistently predicted physical function. Although, this study did not provide a full test of the FAM as no measure of pain catastrophising was included. Therefore, the aim of this study is to replicate the model tested by Swinnen and colleagues, but further extend this research by examining the contribution of both pain catastrophising and fear of movement to the physical function of people living with axSpA in the UK.

### Why is competence frustration important to investigate?

A relevant but untested modifiable predictor of participation in activity for people living with axSpA is perceived competence towards physical activities. In the broader health psychology literature, perceived competence has consistently been shown to support health behaviour change [21, 22], help people adapt to chronic illness [23], identified as an important coping mechanism for chronic pain [24], and positively relates to physical health [25]. Along with perceptions of autonomy and relatedness, within Basic Psychological Needs Theory [21] it is proposed that competence frustration is predictive of human dysfunction. Competence frustration is experienced when one has doubts about their self-efficacy or feels a sense of failure and helplessness towards an activity [26].

Competence frustration appears particularly salient to the FAM as a sense of pain and fear of movement has a direct effect on a person’s sense of ineffectiveness and a sense of helplessness (i.e., they may want to be active but don’t want to cause pain or damage) [27]. Therefore, despite the evidenced and conceptual relevance of competence frustration, little research has examined the role that competence frustration towards participating in activity has in the fear-avoidance model, nor examined the relationship competence frustration has with an indicator of physical function in people living with axSpA.

### Research aims

Given that people living with axSpA report fear as influencing their movement and the importance of activity in the maintenance of physical function for continued quality of life, there are three aims of this research: First, to replicate the mediation analysis conducted by Swinnen et al. [13] in a sample of people living with axSpA in the UK. Second, to extend this model and conduct the initial examination of the theorised contribution of pain catastrophising to axSpA patient’s physical function and the mediating role of fear of movement. Third, to test the additional contribution of competence need frustration to the fear-avoidance model of pain in people living with axSpA. Specifically, we aim to test whether competence frustration and fear of movement mediate the relationship between pain catastrophising and physical function.

## Methods

### Main outcome variable

#### Physical function

The Bath Ankylosing Spondylitis Functional Index (BASFI; [28]) measured patient-report physical function (functional disability). The 10-item scale rates perceived difficulty in performing activities on an 11-point scale (0 = easy, 10 = impossible). A total score was calculated with higher scores indicating worse function. The psychometric properties of the BASFI for people living with axSpA are well established [29].

### Study factors

#### Pain severity

The sum of two items from the 6-item Bath Ankylosing Spondylitis Disease Activity Index (BASDAI; [30], was used as a measure of pain severity [13]. Rated on an 11-point scale (0 = none, 10 = very severe) item 2 measured the overall level of AS neck, back or hip pain and item 3 assessed overall level of pain in joints other than neck, back or hips. The BASDAI has been extensively used as a measure of disease activity, inflammation, and pain in people living with axSpA [31].

#### Pain-related fear of movement

The 11-item Tampa Scale for Kinesiophobia (TSK-11) [32] measured participants fear of pain, movement and (re)injury. Participants respond to each item (e.g., pain lets me know when to stop exercising so that I don’t injure myself) on a 4-point scale ranging from 1 (strongly disagree) to 4 (strongly agree). The 11-item version has been shown to have good construct validity [33].

#### Pain catastrophising

The Pain Catastrophising Scale (PCS) [34] consists of 13-items (e.g., I keep thinking about how much it hurts) that are rated on a scale from 0 (not at all) to 4 (all the time). A total score is calculated (0 to 52) with higher scores indicating greater pain catastrophising. The PCS has previously been employed in a sample of people living with axSpA [19].

#### Competence need frustration

Four items from the Basic Psychological Need satisfaction and Frustration Scale (e.g., I feel like a failure when I participate in physical activity) [35], with the stem adapted to an exercise context, measured participants competence need frustration. All items were rated on a scale from 1 (false) to 6 (true). Higher scores indicated more basic psychological need frustration. Factorial and construct validity related to the items have been provided across four countries [35].

### Procedures

A secondary analysis of an online survey (Ingram et al. Unpublished; Appendix 1) distributed in the UK by the National Axial Spondyloarthritis Society (*n* ≈ 3500; NASS, 2019) via a web-link, webpage, and e-newsletter. The online survey was developed and reviewed by two academics from the University of Bath and a Consultant Rheumatologist. The survey was open between 20.12.20 and 15.04.21 using the Jisc Online Survey platform formerly (Bristol Online Surveys). Demographic characteristics including age, gender, employment status, diagnosis duration, and medication were collected as well as validated psychometric instruments of key variables. A purposive sample was recruited and participants over the age of 18 years were required to read a participant information sheet and tick to indicate informed consent before completing the survey. Ethical approval was granted by the University’s Research Ethics Approval Committee for Health (11/12/2020: EP 19/20 087).

### Statistical analysis

Data were analyzed using SPSS software (Version 28; IBM SPSS Predictive Analytics, Chicago, IL, USA). The distribution of variables was assessed using the Kolmogorov–Smirnov test of normality. Bivariate correlations between the variables were calculated. Correlations of 0.1, 0.3 and 0.5 were considered small, medium and large effects sizes [36]. The PROCESS SPSS macro [37] was used to conduct two mediation analyses and one multiple mediation analysis. Due to the small sample size, mediation analyses were bootstrapped to reduce the risk of Type I errors. Bootstrapping provides a robust method that can be employed with non-normally distributed data and reduces the need for outliers to be identified [38]. As recommended with small sample sizes, 95% percentile bootstrap confidence intervals (95% PBCI) were interpreted from 5000 bootstrap samples [39]. Standardized effects with values 0.01, 0.09, and 0.25 represent small, medium, and large effects [36].

## Results

### Patients

Participants (*N* = 98) were 70% female with a mean age of 45.62 years (SD = 12.16). Seventy-six participants self-reported a diagnosis of axSpA and 22 self-reported a diagnosis of non-radiographic axSpA with a mean diagnosis duration of 11.94 years (SD = 15.95). Of this sample, 49.48% were in full-time employment, 19.59% part-time employment, 8.25% homemaker, 17.53% retired and 5.15% retired due to axSpA. Fifty-nine percent reported that they were currently using Non-Steroidal Anti-Inflammatory Drugs, 62% reported use of Biological Disease-Modifying Anti-Rheumatic Drugs, and 49% were employed full time.

### Bivariate correlations

Means, standard deviations, missing data and bivariate correlations between the variables are shown in Table 1. Bivariate correlations showed that pain, pain catastrophising, fear of movement and competence frustration had significant medium and positive relationships with physical function. Pain catastrophising had a large significant and positive relationship with fear of movement and significant medium and positive relationships with pain and competence frustration. Fear of movement had medium significant positive relationships with pain and competence frustration. Competence frustration had a small positive relationship with pain.

**Table 1:** Means, standard deviations, missing data and bivariate correlations between the variables

	*N*	*α*	Mean	SD	Pain (BASDAI)	Fear of movement	Pain catastrophising	Competence frustration
Physical function (BASFI)	98	0.94	3.73	2.59	0.40**	0.31**	0.41**	0.47**
Pain (BASDAI)	98	0.83	4.62	2.25		0.41**	0.42**	0.26*
Fear of movement	98	0.89	24.99	7.22			0.65**	0.41**
Pain catastrophising	98	0.96	18.34	13.65				0.42**
Competence frustration	95	0.92	3.18	1.54				

*BASFI* Bath Ankylosing Spondylitis Functional Index, *BASDAI* Bath Ankylosing Spondylitis Disease Activity Index

*p < .05, **p < .01

### Mediating tests of the fear-avoidance model

**Fig. 2:**
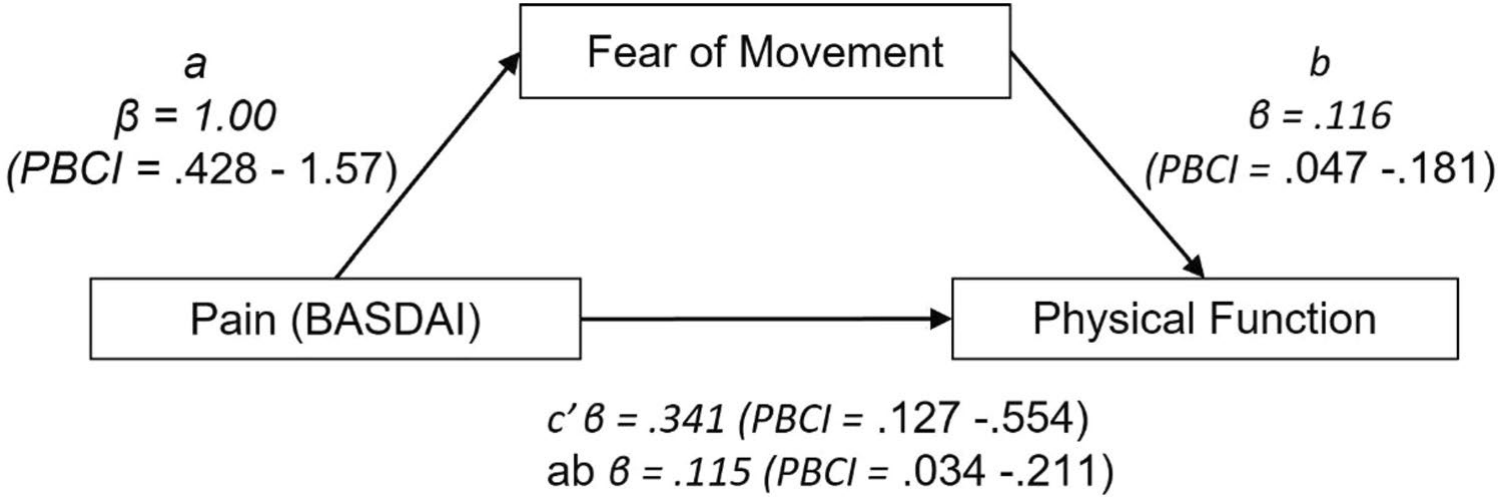
Path model with unstandardised regression coefficients (*B*) and percentile bootstrap confidence intervals (PBCI) identifying the mediating effect of fear of movement in the relationship between pain and physical function. c′ = direct effect, ab = indirect effect

Mediation analysis (*F* = 15.89, *p* < 0.05, *R*^2^ = 0.25) revealed that pain had a large direct effect on physical function (*β* = 0.30), and fear of movement (*β* = 0.31). Fear of movement had a large significant effect on physical function (*β* = 0.32). A significant indirect effect showed the relationship between pain and physical function to be significantly mediated by fear of movement (*β* = 0.10, 95% PBCI = 0.030–0.183; Fig. 2).

**Fig. 3:**
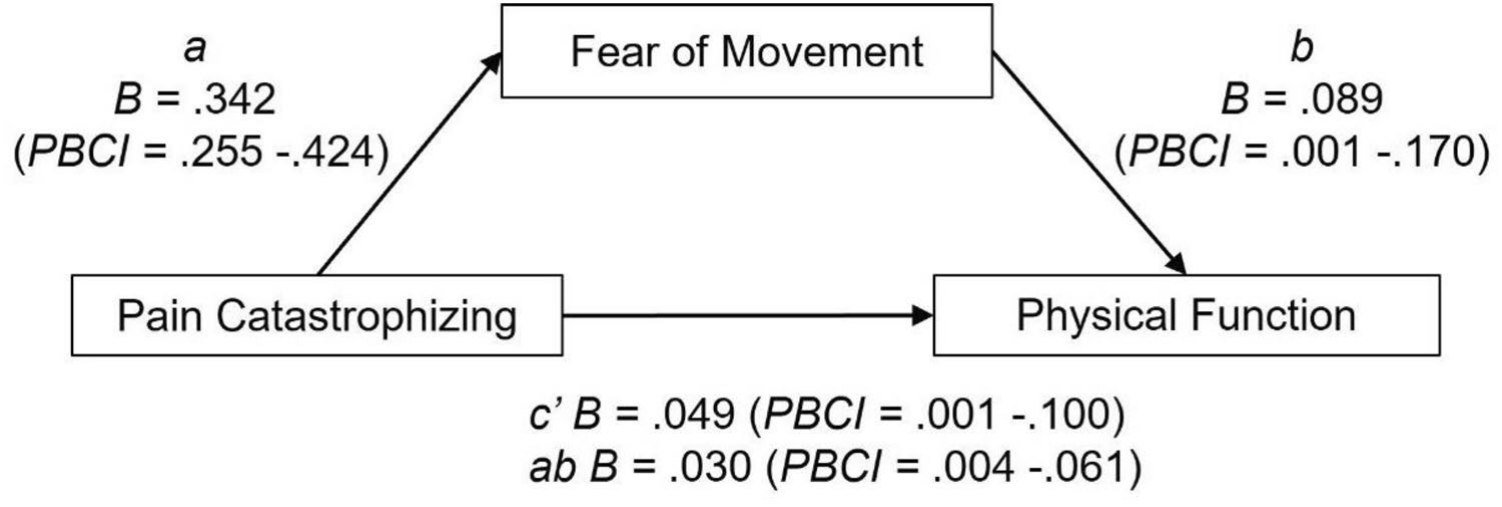
Path model with unstandardised regression coefficients (*B*) and percentile bootstrap confidence intervals (PBCI) identifying the mediating effect of fear of movement in the relationship between pain catastrophising and physical function. c′ = direct effect, ab = indirect effect

Model 2 examined the mediating effect of fear of movement on the relationship between pain catastrophising and physical function. Mediation analysis (*F* = 12.67, *p* < 0.05, *R*^2^ = 0.21) revealed that pain catastrophising had a large direct effect on physical function (*β* = 0.26), and fear of movement (*β* = 0.65). Fear of movement had a moderate significant effect on physical function (*β* = 0.25). A significant indirect effect showed the relationship between pain catastrophising and physical function to be significantly mediated by fear of movement (*β* = 0.16, 95% PBCI = 0.005–0.322; see Fig. 3).

### Mediating contribution of competence frustration

**Fig. 4:**
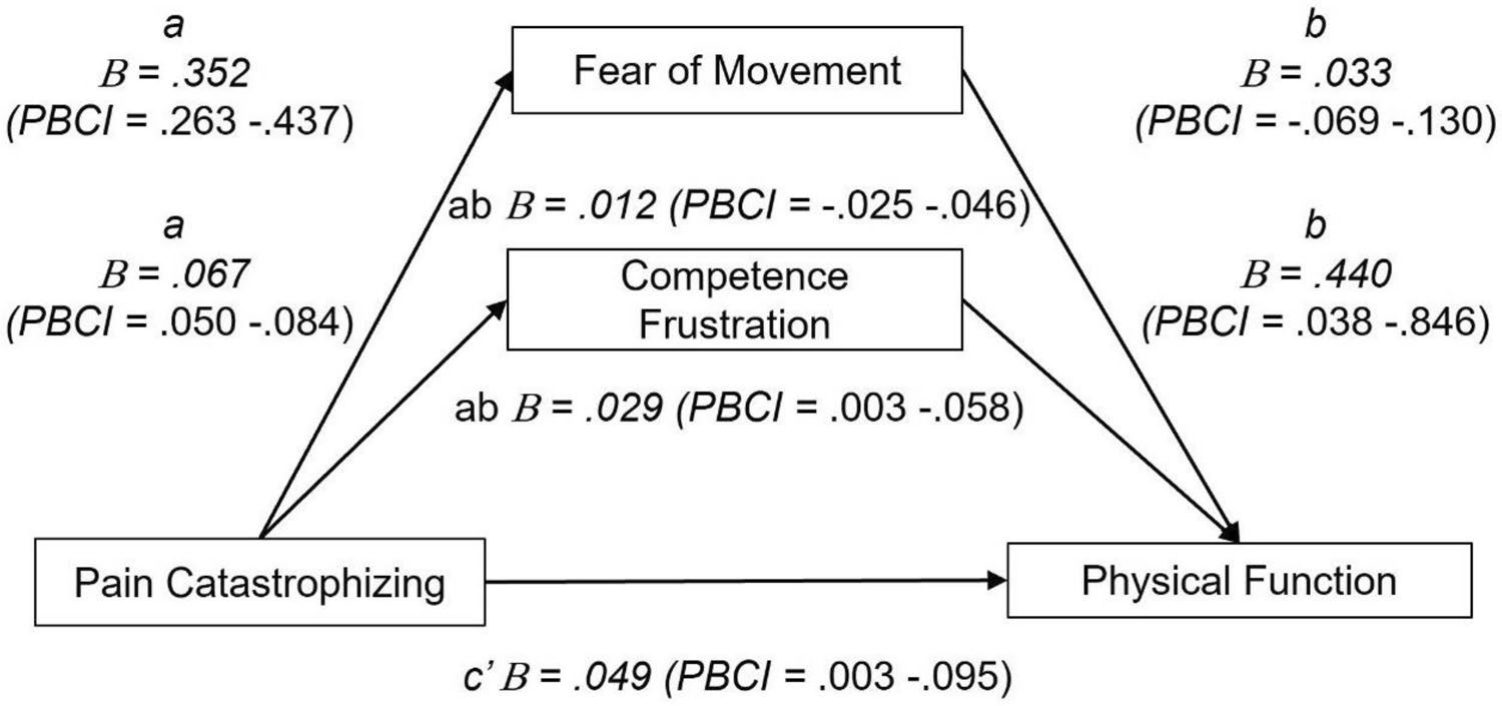
Path model with unstandardised regression coefficients (*B*) and percentile bootstrap confidence intervals (PBCI) identifying the mediating effects of fear of movement and competence frustration in the relationship between pain catastrophising and physical function. c′ = direct effect, ab = indirect effect

Model 3 extended the fear of movement model by examining the unique contribution of competence frustration to the relationship between pain catastrophising and physical function. A multiple mediation analysis (*F* = 12.23, *p* < 0.05, *R*^2^ = 0.29) revealed that pain catastrophising had a large direct effect on physical function (*β* = 0.26), fear of movement (*β* = 0.66) and competence frustration (*β* = 0.59). Fear of movement had a small non-significant effect on physical function (*β* = 0.09) whereas competence frustration had a large significant effect on physical function (*β* = 0.44). A significant indirect effect showed the relationship between pain catastrophising and physical function to be significantly mediated by competence frustration (*β* = 0.15, 95% PBCI = 0.014–0.309) but a non-significant indirect effect indicated that fear of movement did not mediate this relationship (*β* = 0.062, 95% PBCI = *− *0.134 to 0.248; see Fig. 4).

## Discussion

People living with axSpA report experiencing a sense of fear related to movement [13], yet research highlights the importance of activity to maintain physical function [10]. The purpose of this study was to test and extend the fear-avoidance model in a sample of people living with axSpA in two important ways. First, by providing the initial examination of the relationship between pain catastrophising and physical function in a sample of people living with axSpA in the UK, and second, by uniquely adding and testing the mediating role of competence need frustration in the fear-avoidance model. Results revealed the significant negative relationships that both pain-related fear of movement and pain catastrophising make to physical function in people living with axSpA. Subsequently, the findings are novel in identifying the role that competence frustration plays in the fear-avoidance model for people living with axSpA by evidencing that competence need frustration mediates the relationship between pain catastrophising and physical function. Such results highlight the contribution that these modifiable psychological constructs hold for important disease outcomes (e.g., physical function) and provide important and malleable targets for intervention by health professionals.

### Fear-avoidance model

Despite the prominent experience of back pain, it is surprising that limited research has examined the value of the fear-avoidance model for people living with axSpA. Swinnen et al. [13] reported support for the model in a sample of 178 Dutch axSpA patients with their findings showing that fear of movement significantly mediated the relationship between pain and physical function. Our first mediation model replicated the findings of Swinnen and colleagues and provided further support that pain-related fear of movement mediates the relationship between pain and physical function in people living in the UK with axSpA. Yet, Swinnen and colleagues did not examine the role of pain catastrophising, a construct the fear-avoidance model also hypothesises to predict function in the context of pain [40]. Our second model revealed the significant mediating role of fear of movement in the relationship between pain catastrophising and physical function explaining 21% of the variance. Results of the two mediation models provide support for the propositions of the fear-avoidance model and emphasise the important contribution of both pain catastrophising and fear of pain in predicting the physical function of people living with axSpA.

### Competence frustration

Prominent symptoms of axSpA are pain and compromised physical function that can limit both physical movement and perceptions of competence to successfully participate in activity [10, 13]. Basic Psychological Needs Theory (BPNT) [21] proposes that perceptions of competence is one of three innate and universal basic psychological needs that is an essential nutriment for human functioning. When the need for competence is frustrated, BPNT proposes that maladaptive outcomes such as reduced physical activity and compromised human functioning will ensue. This study offers a unique contribution to the literature in this regard, and to the fear-avoidance model, by examining the role that perceptions of competence need frustration play in the prediction of physical function in the context of chronic pain and fear of movement. To our knowledge, this is the first time that the role of competence frustration has been examined in the context of the fear-avoidance model in people living with axSpA. Results of the multiple mediation analysis showed increases in pain catastrophising to be significantly related to increases in fear of movement and competence frustration. Subsequently, and in comparison to the previous two mediation models, fear of movement was not significantly related to poorer physical function whereas increases in competence frustration were significantly associated with poorer physical function. Further, the multiple mediation model revealed that only competence frustration significantly mediated the relationship between pain catastrophising and physical function. Therefore, the effect of pain catastrophising on physical function occurs via an enhanced sense of competence frustration towards participating in physical activities. The prominent role of competence frustration aligns with the propositions of BPNT and that proposed by Riggenbach and colleague’s Developmental Goal Pursuit Model of Pediatric Chronic Pain [41]. Future research should, therefore, investigate how to effectively reduce perceptions of competence frustration and enhance perceptions of competence satisfaction.

### Applied implications

The identification of modifiable psychological constructs that are predictive of established disease outcomes (e.g., physical function, measured via the BASFI), emphasizes the importance of adopting a biopsychosocial approach to care [42]. A biopsychosocial approach supports a holistic understanding of living with a chronic condition that compromises mobility and function. Such evidence highlights the need for health professionals and rehabilitation programmes to show consideration and awareness of a range of biological, psychological, and social factors that contribute to disease outcomes and disease progression. Psychological theories such as Self-determination theory (SDT) and the fear-avoidance model of pain provide frameworks to guide the design and implementation of methods of care. SDT has identified and evidenced a broad range of motivation behaviour change techniques that health professionals can employ to reduce the frustration and support the satisfaction of the basic psychological need for competence [43, 44]. Yet to date, within health contexts, research has focussed on the promotion of competence, there is less research testing specific techniques that alleviate competence frustration. Future research should employ qualitative and experimental research methods to identify techniques that are most effective at creating change in the proposed mechanism of action for physical function in people living with axSpA (i.e., competence frustration and fear of movement).

### Strengths and limitations

The present research carries important applied and theoretical contributions by testing and extending theoretical frameworks to identify modifiable factors that contribute to important disease-related outcomes. Our study has uniquely extended previous research and psychological theory by confirming the relevance and extension of the fear-avoidance model for people living with axSpA in the UK. Yet, there are important limitations to this secondary analyses of a cross-sectional online survey that need consideration when interpreting findings. No causal relationships can be ascertained via our study design, therefore future research employing prospective longitudinal designs to test the direction or reciprocal nature of the relationships identified is warranted. The large percentage of female participants is not representative of the axSpA population; thus future research is also needed to confirm whether these relationships hold true in larger and more gender representative samples. It is also plausible that the length and topic of the survey may have contributed to the small sample size recruited, hence compromising our ability to examine a single comprehensive model that included all the relevant variables. Lastly, other relevant constructs, and confounding variables (e.g., body mass index, depression), that are theorised to contribute to the fear-avoidance model could also be examined such as the basic psychological need for autonomy as well as device measured activity and sedentary behaviour.

## Conclusion

In conclusion, results support the important contribution that the fear-avoidance model makes in predicting physical function, a primary treatment outcome in the management of axSpA. We have also provided support for the integration of the basic psychological needs theory to the fear-avoidance model highlighting the predictive utility of competence need frustration. Future research should aim to test and explore other relevant constructs such as sedentary behaviour/physical activity and examine the sequential path that predicts physical function in people that experience chronic pain.

### Supplementary Information

Below is the link to the electronic supplementary material.

Supplementary file1 (PDF 492 KB)

## Data Availability

The data that support the findings from this research are available from the corresponding author upon reasonable request.
