# Supplementary material for: Fear of movement and competence frustration mediate the relationship between pain catastrophising and physical function in people living with axSpA: an online cross-sectional survey
Source: Rheumatol Int. 2024 Mar 20;44(5):933–41. doi: 10.1007/s00296-024-05557-w (PMC10980646; doi:10.1007/s00296-024-05557-w)
Supplement: Supplementary file 1 — Supplementary file1 (PDF 492 KB) [file 296_2024_5557_MOESM1_ESM.pdf]

Experiences of physical activity in those living with axSpA

Page 1: Participant Information Sheet

Experiences of physical activity in those living with axial spondyloarthritis: a questionnaire survey

Name of Researcher: [redacted]
Email: [redacted]

Name of Supervisor: [redacted]
Email: [redacted]

This information sheet forms part of the process of informed consent. It should give you the basic idea of what the research is about and what your participation will involve. Please read this information sheet carefully and ask one of the researchers named above if you are not clear about any details of the project.

1. What is the purpose of the project:

You are being invited to participate in this online survey by a specialist research team at the University of Bath and we want to better understand your experiences of living with this condition and your experiences of physical activity. The purpose of this survey is to better understand the factors associated with physical activity behaviour and the role physical activity may or may not have in managing the symptoms indicative of axial spondyloarthritis (axSpA). There are no right or wrong answers to these questions; we would just like to understand the normal, everyday experiences of those living with axSpA.

2. Why have I been selected to take part? [or Who can be a participant?]

To participate in this study, you should have a diagnosis of axial spondyloarthritis and be over the age of 18 years.

3. Do I have to take part?

Taking part in this survey is completely voluntary. Before you decide to take part in this project, we will describe the project in this information sheet and give you the opportunity to ask any questions you may have. If agree to take part, we will then ask you to sign a consent form. If at any time you no longer wish to take part in this project, you will be free to withdraw from the study at any point without giving a reason.

4. What will I be asked to do?

You will be asked to answer several questions regarding your experience of physical activity and your current disease activity. The format of the questions ranges from free text entry to multiple choice. The questionnaire is expected to take 30 minutes. You may have experienced some of these questions in clinic before.

5. What are the exclusion criteria? (are there reasons why I should not take part)?

You should not participate in this study if you are under the age of 18 years and/or not able to communicate in English.

6. What are the possible benefits of taking part?

You will be contributing to a research project aiming to understand and improve the lives of those living with SpA, in addition to directing future research looking to maximise the benefits of physical activity.

7. What are the possible disadvantages and risks of taking part?

There are no disadvantages or risks associated with taking part in this project. If there are questions that you do not feel comfortable answering for any reason, you may choose not to provide an answer.

8. Will my participation involve any discomfort or embarrassment?

We do not expect you to feel any discomfort or embarrassment if you take part in this project. However, if any of the questions do cause you to feel any discomfort, you may choose not to answer them or you may withdraw from the study entirely without reason. At the end of the survey we also direct you to an appropriate support service.

9. Who will have access to the information that I provide?

Only the research team working on this project will have access to the information you provide. All records will be treated confidential.

10. What will happen to the data collected and results of the project?

The information we collect will only be used for research purposes. All collected data will be treated as confidential and stored on a password protected file on the University of Bath's secure server (X drive). To safeguard your rights, we will use the minimum personally-identifiable information possible.

All data from the survey is anonymous as no identifiable information is taken unless you indicate the following. If at the end of the survey you have indicated a willingness to complete the survey again in a years' time or receive a summary of the findings then you will have provided us with a name and email address. This name and email address will be linked to your individual study number ID. The survey data will be immediately detached from your name and email address and stored on a protected file the University of Bath's secure server. The document containing your contact details will be deleted once the summary of findings and/or follow-up survey has been completed.

Any information that could identify you will be held securely with strict arrangements about who can access the information. We will retain the study data for 10 years after the study has finished, and it will be stored in accordance with the GDPR and the Data Protection Act.

The information you provide will be confidential and anonymous, thus no identifiable information will be included in any published reports, conferences, or scientific journals. Participants may opt in to receive a summary of the findings from this project, which will also not contain any identifiable information.

11. Who has reviewed the project?

This project has been given a favourable opinion by the University of Bath, Research Ethics Approval Committee for Health (REACH) [reference: EP 19/20 087].

12. How can I withdraw from the project?

Your rights to access, change or move your information are limited, as we need to manage your information in specific ways in order for the research to be reliable and accurate. It will not be possible to withdraw your anonymous data after the survey has been completed, however this may be possible before the data analysis stage for individuals who have provided us with an email address. If you exit the survey before completion, no data will be stored

13. University of Bath privacy notice

The University of Bath privacy notice can be found here: https://www.bath.ac.uk/corporate-information/university-of-bath-privacy-notice-for-research-participants/

14. What happens if there is a problem?

If you have any concerns about any aspect of the project, please contact one of the researchers (information provided above) who will do their best to answer your questions. If they are not able to help, or you wish to make a complaint regarding the project, please contact [redacted] (Chair of the Research Ethics Approval Committee for Health) via email [redacted]

15. If I require further information who should I contact and how?

Thank you for expressing an interest in participating in this project. Please do not hesitate to get in touch with us if you would like some more information.

Name of Researcher: [redacted]

Email: [redacted]

Name of Supervisor: [redacted]

Email: [redacted]

Page 2: Consent Form

Please initial each of the following boxes if you agree with the statement:

1. I have been provided with the information explaining what participation in this project involves. ✱ *Required*

2. I have had an opportunity to ask questions and discuss this project. ✱ *Required*

3. I have received satisfactory answers to all questions I have asked. ✱ *Required*

4. I have received enough information about the project to make a decision about my participation. ✱ *Required*

5. I understand that I am free to withdraw my consent to participate in the project at any time without having to give a reason for withdrawing. ✱ *Required*

6. I understand that I am free to withdraw my data up to the completion of the survey. ✱ *Required*

7. I understand the nature and purpose of the procedures involved in this project. These have been communicated to me on the information sheet accompanying this form. ✱ *Required*

8. I understand and acknowledge that the investigation is designed to promote scientific knowledge and that the University of Bath will

use the data I provide only for the purpose(s) set out in the information sheet. If I indicate a willingness to complete the one year's follow-up the conditions on this form under which I have provided the data will still apply. ✱ *Required*

9. I understand the data I provide will be treated as confidential, and that on completion of the project my name or other identifying information will not be disclosed in any presentation or publication of the research. ✱ *Required*

10. I agree to the University of Bath keeping and processing the data that I provide during the course of this project and my consent is conditional upon the University complying with its duties and obligations under the Data Protection Act. ✱ *Required*

11. I hereby fully and freely consent to my participation in this project. ✱ *Required*

Page 3: Demographic Information

12. Age (years):

[ ]

13. Gender:

- ○ Male
- ○ Female
- ○ Non-binary
- ○ Prefer not to say
- ○ Other

14. Is your gender identity different from the gender you were assigned at birth?

- ○ Yes
- ○ No
- ○ Prefer not to say

15. Type of Axial Spondyloarthritis (axSpA) you have:

- ○ Non-radiographic Axial Spondyloarthritis
- ○ Ankylosing Spondylitis

16. Number of years with axSpA symptoms:

[ ]

17. Number of years with axSpA diagnosis:

[ ]

18. Current use of Non-Steroidal Anti-Inflammatory Drugs:

- ○ Yes
- ○ No

19. Current use of Biological Therapies:

- Yes
- No

20. Employment status:

- Full-time employment
- Part-time employment
- Homemaker
- Retired
- Retired due to axSpA disability

21. If you are currently in employment, do you have a desk job or spend large periods of time sitting?

- Yes
- No
- NA

Page 4: Disease Related

22. Name ONE area of the body (e.g., lower back, ribs, neck, hips, etc) you have the most difficulty with (i.e., pain, discomfort).

23. Do you suffer with morning stiffness?

- Yes
- No

24. If so, do you have difficulty getting out of bed?

- Yes
- No
- NA (no morning stiffness)

25. If so, do you do anything or use anything to combat morning stiffness?

26. During the past week, how many hours sleep did you get per night?

27. During the past week, how would you rate your seep quality?

- Bad
- Poor
- Fair
- Good
- Excellent

28. If you have a BASMI conducted (spinal measurement scored out of 10), how often do you have one?

- > yearly
- Yearly
- <year
- Don't know
- NA

29. Have you ever received physical activity advice from your GP?

- ○ Yes
- ○ No

30. Do you use an activity tracker? (i.e., to measure step counts, physical activity etc.)

- ○ Yes
- ○ No

31. Do you use a smartphone application to track or manage your disease?

- ○ Yes
- ○ No

Page 5: Disease Activity

32. Please indicate which represents your answer. All questions refer to LAST WEEK.

Please don't select more than 1 answer(s) per row.

	0 (None)	1	2	3	4	5	6	7	8	9	10 (Very severe)
1. How would you describe the overall level of fatigue/tiredness you have experienced?	☐	☐	☐	☐	☐	☐	☐	☐	☐	☐	☐
2. How would you describe the overall level of AS neck, back or hip pain you have had?	☐	☐	☐	☐	☐	☐	☐	☐	☐	☐	☐
3. How would you describe the overall level of pain/swelling in joints other than neck, back or hips you have had?	☐	☐	☐	☐	☐	☐	☐	☐	☐	☐	☐
4. How would you describe the overall level of discomfort you have had from any areas tender to touch or pressure?	☐	☐	☐	☐	☐	☐	☐	☐	☐	☐	☐
5. How would you describe the overall level of morning stiffness you have had from the time you wake up?	☐	☐	☐	☐	☐	☐	☐	☐	☐	☐	☐

33. How long does your morning stiffness last from the time you wake up?

Please don't select more than 1 answer(s) per row.

	0 (0 hours)	1	2	3	4	5 (1 hour)	6	7	8	9	10 (2 or more hours)
Scale	☐	☐	☐	☐	☐	☐	☐	☐	☐	☐	☐

34. How would you describe the overall intensity of pain you have experienced in the last 24hrs?

Please don't select more than 1 answer(s) per row.

	0 (no pain)	1	2	3	4	5 (moderate pain)	6	7	8	9	10 (worst possible pain)
Scale	☐	☐	☐	☐	☐	☐	☐	☐	☐	☐	☐

The BASDAI questionnaire is being used with permission from the Royal National Hospital for Rheumatic Diseases, Bath.

Page 6: Functional Limitation

35. Please indicate your level of ability with each of the following activities during the PAST WEEK.

Please don't select more than 1 answer(s) per row.

	0 (None)	1	2	3	4	5	6	7	8	9	10 (Impossible)
1. Putting on your socks or tights without help or aids (e.g., sock aid)	☐	☐	☐	☐	☐	☐	☐	☐	☐	☐	☐
2. Bending from the waist to pick up a pen from the floor without aid	☐	☐	☐	☐	☐	☐	☐	☐	☐	☐	☐
3. Reaching up to a high shelf without help or aids (e.g., helping hand)	☐	☐	☐	☐	☐	☐	☐	☐	☐	☐	☐
4. Getting up from an armless chair without your hands or any other help	☐	☐	☐	☐	☐	☐	☐	☐	☐	☐	☐
5. Getting up off the floor without help from lying on your back	☐	☐	☐	☐	☐	☐	☐	☐	☐	☐	☐
6. Standing unsupported for 10 minutes without discomfort	☐	☐	☐	☐	☐	☐	☐	☐	☐	☐	☐
7. Climbing 12-15 steps without using a handrail or walking aid	☐	☐	☐	☐	☐	☐	☐	☐	☐	☐	☐
8. Looking over your shoulder without turning your body	☐	☐	☐	☐	☐	☐	☐	☐	☐	☐	☐

9. Doing physically demanding activities (e.g., physiotherapy exercises, gardening or sports)	☐	☐	☐	☐	☐	☐	☐	☐	☐	☐	☐
10. Doing a full days activities whether it be at home or at work	☐	☐	☐	☐	☐	☐	☐	☐	☐	☐	☐

The BASFI questionnaire is being used with permission from the Royal National Hospital for Rheumatic Diseases, Bath.

Page 7: How do you view your condition

36. This is a list of phrases which other patients have used to express how they view their condition. Please circle the number that best describes how you feel about each statement

Please don't select more than 1 answer(s) per row.

	Strongly Disagree	Somewhat Disagree	Somewhat Agree	Strongly Agree
1. I'm afraid I might injure myself if I exercise	☐	☐	☐	☐
2. If I were to try to overcome it, my pain would increase	☐	☐	☐	☐
3. My body is telling me I have something dangerously wrong	☐	☐	☐	☐
4. People aren't taking my medical condition seriously enough	☐	☐	☐	☐
5. My problem has put my body at risk for the rest of my life	☐	☐	☐	☐
6. Pain always means I have injured my body	☐	☐	☐	☐
7. Simple being careful that I do not make any unnecessary movements is the safest thing I can do to prevent my pain from worsening	☐	☐	☐	☐
8. I wouldn't have this much pain if there wasn't something potentially dangerous going on in my body	☐	☐	☐	☐
9. Pain lets me know when to stop exercising so that I don't injure myself	☐	☐	☐	☐
10. I can't do all the things normal people do because it's too easy for me to get injured	☐	☐	☐	☐
11. No one should have to exercise when he/she is in pain	☐	☐	☐	☐

Permission to use the TSK-11 was granted by Professor Steve Woby.

Page 8: Pain Experience

Everyone experiences painful situations at some point in their lives. Such experiences may include headaches, tooth pain, joint or mucle pain. People are often exposed to situations that may cause pain such as illness, injury, dental procedures or surgery.

We are interested int he types of thoughts and feelings that you have when you are in pain. Listed below are thirteen statements describing thoughts and feelings that may be associated with pain. Using the following scale, please indicate the degree to which you have these thoughts and feelings when you are experiencing pain.

37. When I'm in pain ...

Please don't select more than 1 answer(s) per row.

	0 - not at all	1 - to a slight degree	2 - to a moderate degree	3 - to a great degree	4 - all the time
1. I worry all the time about whether the pain will end.	☐	☐	☐	☐	☐
2. I feel I can't go on.	☐	☐	☐	☐	☐
3. It's terrible and I think it's never going to get any better.	☐	☐	☐	☐	☐
4. It's awful and I feel that it overwhelms me.	☐	☐	☐	☐	☐
5. I feel I can't stand it anymore.	☐	☐	☐	☐	☐
6. I become afraid that the pain will get worse.	☐	☐	☐	☐	☐
7. I keep thinking of other painful events.	☐	☐	☐	☐	☐
8. I anxiously want the pain to go away.	☐	☐	☐	☐	☐
9. I can't seem to keep it out of my mind.	☐	☐	☐	☐	☐
10. I keep thinking about how much it hurts.	☐	☐	☐	☐	☐
11. I keep thinking about how badly I want the pain to stop.	☐	☐	☐	☐	☐
12. There's nothing I can do to reduce the intensity of the pain.	☐	☐	☐	☐	☐
13. I wonder whether something serious may happen.	☐	☐	☐	☐	☐

PCS - United Kingdom/English - Version of 28 Feb 17 - Mapi. Copyright © 1995 Michael JL Sullivan. All right reserved

Page 9: Physical Activity

We are interested in finding out about the kinds of physical activities that people do as part of their everyday lives. The questions wil ask you about the time you spend being physically active in the last 7 days. Please answer each question even if you do not consider yourself to be an active person. Please think about the activities you do at work, as part of your house and yard work, to get from place to place, and in your spare time for recreation, exercise or sport.

Think about all the **vigorous** activities that you did in the last 7 days. **Vigorous** physical activities refer to activities that take hard physical effort and make you breathe much harder than normal. Think only about those activities that you did for at least 10 minutes at a time.

1. During the last 7 days, on how many days did you do **vigorous** physical activities like heavy lifting, digging, aerobics, or fast bicycling?

38. Days per week:

No **vigorous** physical activities, Skip to question 3

2. How much time did you usually spend doing **vigorous** physical activities on one of those days?

39. Hours per day:

39.a. Minutes per day:

Think about all the **moderate** activities that you did in the last 7 days. **Moderate** actvities refer to activities that take moderate physical effort and make you breathe somewhat harder than normal. Think only about those physical activities that you did for at least 10 minutes at a time.

3. During the last 7 days, on how many days did you do **moderate** physical activities like carrying light loads, bicycling at a regular pace, or doubles tennis? Do no include walking.

40. Days per week:

No **moderate** physical activities, Skip to question 5

4. How much time did you usually spend doing **moderate** physical activities on one of those days?

41. Hours per day:

41.a. Minutes per day:

Think about the time you spent **walking** in the last 7 days. This includes at work and at home, **walking** to travel from place to place, and any other **walking** that you have done solely for recreation, sport, exercise, or leisure.

5. During the last 7 days, on how many days did you **walk** for at least 10 minutes at a time?

42. Days per week:

No **walking**, Skip to question 7

6. How much time did you usually spend **walking** on one of those days?

43. Hours per day:

43.a. Minutes per day:

The last question is about the time you spent **sitting** on weekdays during the last 7 days. Include time spent at work, at home, while doing course work and during leisure time. This may include time spent **sitting** at a desk, visiting friends, reading, or **sitting** or lying down to watch television.

7. During the last 7 days, how much time did you spend **sitting** on a week day?

44. Hours per day:

44.a. Minutes per day:

Page 10: Well-being

45. Please respond to each of the following statements by indicating the degree to which the statement is true for you, in overall, **over the last week**.

Please don't select more than 1 answer(s) per row.

	1 (not at all true)	2	3	4 (somewhat true)	5	6	7 (Very true)
1. I feel alive and full of vitality	☐	☐	☐	☐	☐	☐	☐
2. I have energy and spirit	☐	☐	☐	☐	☐	☐	☐
3. I look forward to each new day	☐	☐	☐	☐	☐	☐	☐
4. I nearly always feel alert and awake	☐	☐	☐	☐	☐	☐	☐
5. I feel I have a lot of energy	☐	☐	☐	☐	☐	☐	☐

Page 11: Encounters with your physician ...

46. This questionnaire contains items that are related to your visits with your rheumatologist. Physicians (rheumatologists) have different styles in dealing with patients, and we would like to know more about how you have felt about your encounters with your rheumatologist. Your responses are confidential. Please be honest and candid.

Please don't select more than 1 answer(s) per row.

	1 (strongly disagree)	2	3	4 (neutral)	5	6	7 (strongly agree)
1. I feel that my physician/consultant had provided me choices and options	☐	☐	☐	☐	☐	☐	☐
2. I feel understood by my physician	☐	☐	☐	☐	☐	☐	☐
3. I am able to be open with my physician at our meetings	☐	☐	☐	☐	☐	☐	☐
4. My physician conveys confidence in my ability to make changes	☐	☐	☐	☐	☐	☐	☐
5. I feel that my physician accepts me	☐	☐	☐	☐	☐	☐	☐
6. My physician has made sure I really understand about my condition and what I need to do	☐	☐	☐	☐	☐	☐	☐
7. My physician encourages me to ask questions	☐	☐	☐	☐	☐	☐	☐
8. I feel a lot of trust in my physician	☐	☐	☐	☐	☐	☐	☐
9. My physician answers my questions fully and carefully	☐	☐	☐	☐	☐	☐	☐
10. My physician listens to how I would like to do things	☐	☐	☐	☐	☐	☐	☐
11. My physician handles people's emotions very well	☐	☐	☐	☐	☐	☐	☐
12. I feel that my physician cares about me as a person	☐	☐	☐	☐	☐	☐	☐
13. I don't feel very good about the way my physician talks to me	☐	☐	☐	☐	☐	☐	☐
14. My physician tries to understand how I see things before suggesting a new way to do things	☐	☐	☐	☐	☐	☐	☐
15. I feel able to share my feelings with my physician	☐	☐	☐	☐	☐	☐	☐

Page 12: Important Other

Who (partner, best friend, offspring, sibling etc) is the most important person in your effort to becoming healthier through regular physical activity?

Please select one important other.

47. My important other:

48. Please answer the questions with respect to that individual.

Please don't select more than 1 answer(s) per row.

	1 (strongly disagree)	2	3	4	5	6	7 (strongly agree)
1. I feel that my important other provided me with choices and options about physical activity and health							
2. I feel my important other understands how I see things with respect to my physical activity and health							
3. My important other conveys confidence in my ability to make changes regarding my physical activity and health							
4. My important other listens to how I would like to do things regarding my physical activity and health							
5. My important other encourages me to ask questions about my physical activity to improve my health							
6. My important other tries to understand how I see my health-related physical activity before suggesting changes							

Page 13: Experiences of physical activity

49. The following statements represent different experiences people have when they exercise or engage in physical activity. Please answer the following questions by considering how **YOU TYPICALLY** feel while you are exercising/ engaging in physical activity.

Please don't select more than 1 answer(s) per row.

	1 (False	2 (Mostly false)	3 (More false than true)	4 (More true than false)	5 (Mostly true)	6 (True)
1. I feel that I am able to participate in physical activities that are personally challenging	☐	☐	☐	☐	☐	☐
2. I feel attached to those who participate in physical activities with me because they accept me for who I am	☐	☐	☐	☐	☐	☐
3. I feel like I share a common bond with people who are important to me when we participate in physical activities together / exercise together	☐	☐	☐	☐	☐	☐
4. I feel confident I can do even the most challenging exercises / physical activities	☐	☐	☐	☐	☐	☐
5. I feel a sense of camaraderie with those people I am active with because we engage in physical activity for the same reasons	☐	☐	☐	☐	☐	☐
6. I feel confident in my ability to perform exercises / physical activities that personally challenge me	☐	☐	☐	☐	☐	☐
7. I feel close to those I am physically active with as they appreciate how difficult regular engagement in physical activity can be	☐	☐	☐	☐	☐	☐
8. I feel free to be physically active in my own way	☐	☐	☐	☐	☐	☐
9. I feel free to make my own decisions regarding my participation in physical activity	☐	☐	☐	☐	☐	☐
10. I feel capable of doing physical activities that are challenging to me	☐	☐	☐	☐	☐	☐
11. I feel like I am in charge of my exercise program decisions	☐	☐	☐	☐	☐	☐
12. I feel like I am capable of doing even the most challenging physical activities	☐	☐	☐	☐	☐	☐
13. I feel like I have a say in choosing the exercises / physical activities that I do	☐	☐	☐	☐	☐	☐
14. I feel connected to the people who I interact with while we participate in physical activity together	☐	☐	☐	☐	☐	☐
15. I feel good about the way I am able to complete challenging exercises / physical activities	☐	☐	☐	☐	☐	☐
16. I feel like I get along well with other people who I interact with while we are physically active together	☐	☐	☐	☐	☐	☐
17. I feel free to choose which exercises / physical activities I participate in	☐	☐	☐	☐	☐	☐
18. I feel like I am the one who decides what exercises I do	☐	☐	☐	☐	☐	☐

Page 14: More Experiences of Physical Activity

50. The following statements represent difference experiences people have when they exercise or engage in physical activity. Please answer the following questions by considering how **YOU TYPICALLY** feel while you are exercising / engaging in physical activity.

Please don't select more than 1 answer(s) per row.

	1 (False)	2 (Mostly false)	3 (More false than true)	4 (More true than false)	5 (Mostly true)	6 (True)
1. I feel like I have to participate in exercise	☐	☐	☐	☐	☐	☐
2. I feel forced to do exercises / physical activities I wouldn't choose to do	☐	☐	☐	☐	☐	☐
3. I feel pressured to participate in physical activities	☐	☐	☐	☐	☐	☐
4. I feel obliged to participate in physical activities	☐	☐	☐	☐	☐	☐
5. I seriously doubt that I can be successful at physically active tasks	☐	☐	☐	☐	☐	☐
6. I feel disappointed with my attempts to be physically active	☐	☐	☐	☐	☐	☐
7. I feel insecure about my ability to be physically active	☐	☐	☐	☐	☐	☐
8. I feel like a failure when I participate in physical activity	☐	☐	☐	☐	☐	☐
9. I feel excluded by the people I exercise with	☐	☐	☐	☐	☐	☐
10. The people I exercise with are cold and distant towards me	☐	☐	☐	☐	☐	☐
11. The people I exercise with dislike me	☐	☐	☐	☐	☐	☐
12. I have superficial relationships with the people I exercise with	☐	☐	☐	☐	☐	☐

Page 15: Your Reasons For Engaging In Physical Activity

51. We are interested in the reasons underlying people's decisions to engage or not engage in current physical activity / exercise. Using the scale below, please indicate to what extent each of the following items is true for you.

Please don't select more than 1 answer(s) per row.

	0 (Not at all true)	1	2 (Somewhat true)	3	4 (Very true)
1. I engage in physical activity because other people say I should	☐	☐	☐	☐	☐
2. I feel guilty when I don't exercise	☐	☐	☐	☐	☐
3. I value the benefits of physical activity	☐	☐	☐	☐	☐
4. I engage in physical activity because it's fun	☐	☐	☐	☐	☐
5. I don't see why I should have to be physically active	☐	☐	☐	☐	☐
6. I take part in physical activity because my friends/family/partner say I should	☐	☐	☐	☐	☐
7. I feel ashamed when I miss an exercise session / chance to be physically active	☐	☐	☐	☐	☐
8. It's important for me to regularly participate in physical activity	☐	☐	☐	☐	☐
9. I can't see why I should bother being physically active	☐	☐	☐	☐	☐
10. I enjoy my exercise sessions / participation in physical activity	☐	☐	☐	☐	☐
11. I engage in physical activity because others will not be pleased with me if I don't	☐	☐	☐	☐	☐
12. I don't see the point in being physically active	☐	☐	☐	☐	☐
13. I feel like a failure when I haven't been physically active in a while	☐	☐	☐	☐	☐
14. I think it is important to make the effort to regularly participate in physical activity	☐	☐	☐	☐	☐
15. I find physical activity pleasurable	☐	☐	☐	☐	☐
16. I feel under pressure from my friends/family to participate in physical activity	☐	☐	☐	☐	☐

17. I get restless if I don't regularly participate in physical activity	☐	☐	☐	☐	☐
18. I get pleasure and satisfaction from participating in physical activity	☐	☐	☐	☐	☐
19. I think engaging in physical activity is a waste of time	☐	☐	☐	☐	☐

Page 16: Stretching

52. Do you conduct stretches for your condition?

- Yes
- No

52.a. If so, do you allocate specific time in the day to conduct them?

- Yes
- No

52.b. Or, do you incorporate them into your daily activities (e.g., when making a cup of tea)?

- Yes
- No

52.c. If you stretch, how long would you spend stretching in a single bout?

53. In the last 7 days, how many days did you conduct stretches?

- 0
- 1
- 2
- 3
- 4
- 5
- 6
- 7

53.a. In the last 7 days, how long on average did you spend stretching each day?

54. Have you been advised or trained how to conduct stretches correctly?

- Yes
- No

54.a. If so, what source did you learn from?

55. For each of the following statements, please indicate how true it is for you regarding STRETCHING.

Please don't select more than 1 answer(s) per row.

	1 (Not at all true)	2	3	4 (Somewhat true)	5	6	7 (Very true)

1. I didn't put much energy into stretching							
2. While I was working on stretching I was thinking about how much I enjoyed it							
3. I did not feel at all nervous about stretching							
4. I put a lot of effort into stretching							
5. I believe stretching could be of some value to me							
6. I believe I had some choice about doing stretching							
7. I think I am pretty good at stretching							
8. Stretching did not hold my attention at all							
9. I think this is important to do because it can help manage my condition							
10. I felt tense while doing stretching							
11. I think I did pretty well at stretching, compared to others							
12. Stretching was fun to do							
13. I didn't try very hard to do well at stretching							
14. I think stretching is an important activity							
15. I felt relaxed while doing stretching							
16. I did stretching because I had to							
17. I think that stretching is useful for my condition							
18. I enjoyed doing stretching very much							
19. I didn't really have a choice about doing stretching							
20. I am satisfied with my performance at stretching							
21. I tried very hard on stretching							
22. I was nervous while doing stretching							
23. I felt it was not my choice to do stretching							
24. I think doing stretching could help to to manage my condition							
25. I thought stretching was very boring							
26. I do stretching because I wanted to							
27. I felt pretty skilled at stretching							
28. This was an activity that I couldn't do very well							

29. I would describe stretching as very interesting	☐	☐	☐	☐	☐	☐	☐
30. I felt pressured while doing stretching	☐	☐	☐	☐	☐	☐	☐
31. I would be willing to do this again because it has some value to me	☐	☐	☐	☐	☐	☐	☐
32. I felt like I had to do stretching	☐	☐	☐	☐	☐	☐	☐
33. I would describe stretching as very enjoyable	☐	☐	☐	☐	☐	☐	☐
34. I believe doing stretching could be beneficial to me	☐	☐	☐	☐	☐	☐	☐
35. I did stretching because I had no choice	☐	☐	☐	☐	☐	☐	☐
36. After working at stretching for a while, I felt pretty competent	☐	☐	☐	☐	☐	☐	☐
37. It was important to me to do well at stretching	☐	☐	☐	☐	☐	☐	☐

Page 17: Further Information

56. Would you be willing to be contacted and complete this questionnaire in a years' time?

- Yes
- No
- Maybe

If yes or maybe, please provide your contact details below.

57. Name:

57.a. Email address:

58. Would you like to receive a summary of the findings once the study has been completed? If so, please leave your email address below.

- Yes
- No

58.a. Email address:

Page 18: Final page

The research team at the University of Bath would like to thank you for taking the time to complete this survey.

If you have experienced any discomfort while completing this survey and feel you need extra support, please feel free to contact the NASS Helpline. The NASS Helpline is open Monday to Friday 10am to 4pm. The NASS Helpline telephone number is 020 8741 1515.

The website link below also describes other ways of contacting the helpline including email and various social media accounts.

https://nass.co.uk/contact-us/
